# The Role of Interferon Regulatory Factor-1 (IRF1) in Overcoming Antiestrogen Resistance in the Treatment of Breast Cancer

**DOI:** 10.4061/2011/912102

**Published:** 2011-07-03

**Authors:** J. L. Schwartz, A. N. Shajahan, R. Clarke

**Affiliations:** Georgetown University Medical Center, W401 Research Building, 3970 Reservoir Road, NW, Washington, DC 20057, USA

## Abstract

Resistance to endocrine therapy is common among breast cancer patients with estrogen receptor alpha-positive (ER+) tumors and limits the success of this therapeutic strategy. While the mechanisms that regulate endocrine responsiveness and cell fate are not fully understood, interferon regulatory factor-1 (IRF1) is strongly implicated as a key regulatory node in the underlying signaling network. IRF1 is a tumor suppressor that mediates cell fate by facilitating apoptosis and can do so with or without functional p53. Expression of IRF1 is downregulated in endocrine-resistant breast cancer cells, protecting these cells from IRF1-induced inhibition of proliferation and/or induction of cell death. Nonetheless, when IRF1 expression is induced following IFN**γ** treatment, antiestrogen sensitivity is restored by a process that includes the inhibition of prosurvival BCL2 family members and caspase activation. These data suggest that a combination of endocrine therapy and compounds that effectively induce IRF1 expression may be useful for the treatment of many ER+ breast cancers. By understanding IRF1 signaling in the context of endocrine responsiveness, we may be able to develop novel therapeutic strategies and better predict how patients will respond to endocrine therapy.

## 1. Introduction of Breast Cancer and Antiestrogens

The American Cancer Society estimates that in 2010, 207,090 women were diagnosed with invasive breast cancer, and approximately 40,000 women died from the disease in the United States alone [1]. Despite the advances in treatment, earlier detection through screening, and increased awareness, breast cancer remains the second most diagnosed cancer in women [2]. Approximately 70% of tumors will be estrogen receptor-*α* positive (ER+). Randomized trials and large meta-analyses clearly indicate that all breast cancer patients derive a statistically significant survival benefit from endocrine therapy and chemotherapy [3], with the antiestrogen (AE) tamoxifen (TAM) being among the most effective single agents. This survival benefit reflects the ability of these agents to drive cells down an irreversible cell death pathway [4]. Yet, advanced ER+ breast cancer remains an incurable disease, and improvements in overall survival rates for these women have been relatively modest during this timeframe [5].

Endocrine therapy is generally administered as an antiestrogen, such as TAM or fulvestrant (FAS; Faslodex; ICI 182,780), or as an aromatase inhibitor (AI) including letrozole or exemestane. TAM is the most frequently used AE and significantly reduces the risk of recurrence and death in patients with ER+ disease [6]. Five years of TAM is a standard treatment for both premenopausal and postmenopausal women with ER+ breast cancer [7]. Mechanistically, TAM is a selective estrogen receptor modulator (SERM) that competes with the endogenous ER substrate (17*β*-estradiol) for binding to the receptor protein [8]. TAM induces a conformational change in the receptor that, in turn, represses ER transcriptional activity [9]. While TAM has been the treatment of choice for over three decades, third-generation AIs have demonstrated a greater disease-free benefit than TAM and are often now the first line endocrine therapy of choice for postmenopausal women with ER+ breast cancer [10]. Newer antiestrogens, such as the selective estrogen receptor down regulator fulvestrant, do not exhibit the partial agonist activities of SERMs and are effective treatment options after relapse on TAM [11]. FAS has been shown to be as effective as the AI anastrozole in postmenopausal women with advanced breast cancer resistant to TAM [12].

## 2. Endocrine Resistance

Despite AIs and AEs being key modalities in the treatment of ER+ breast cancers, the inability of these therapies to cure all women with ER+ disease remains a major challenge to the clinical and research communities [13]. In the case of TAM, *de novo* (intrinsic) or acquired resistance limits the efficacy of treatment in many patients with ER+ disease. Approximately 50% of ER+ tumors are *de novo *resistant. Moreover, some tumors may become resistant to endocrine therapies despite the continued presence of ER; a small proportion may also become ER-negative (ER−). Consequently, we fail to predict responsiveness to endocrine therapy in 66% of ER+/progesterone receptor (PgR) negative, 55% of ER−/PgR+, and 25% of ER+/PgR+ tumors [8]. Clearly, better predictors of endocrine responsiveness are required. 

Most TAM-resistant tumors retain detectable levels of ER expression, and many of these will respond to second or third line hormonal therapies [9]. For patients with advanced breast cancer, FAS is beneficial following progression on TAM therapy [14]. Currently, FAS is the only FDA approved drug to treat advanced breast cancer in postmenopausal women who previously progressed on other antiestrogens. However, a significant number of patients will also develop resistance to FAS [15]. The inability to fully modify these negative outcomes reflects an incomplete understanding of the signaling events effecting cell proliferation, survival, and hormonal regulation. Estrogen independence and AE resistance are complex phenotypes, and it is unlikely that a single gene/signaling pathway drives endocrine resistance in ER+ tumors.

Drug resistance arises from a cell's inability to induce signaling down an irreversible cell death pathway. Therefore, understanding the key signaling aspects of resistance and how they are balanced, ultimately leading to cell death/survival, is an important research goal [13]. Several genes associated with survival despite antiestrogen treatment have been identified from gene expression microarrays between antiestrogen-responsive (MCF7/LCC1) and—resistant variants (MCF7/LCC9) of the MCF7 human breast cancer cell line [16]. One transcription factor that is vital in the cell death/survival regulatory network is interferon regulatory factor-1 (IRF1), which is downregulated in the resistant cell line. These data and others suggest that the downregulation of IRF1 protects breast cancer cells from IRF1-induced inhibition of cell proliferation and/or induction of apoptosis [16, 17]. In the present review paper, we summarize the role of IRF1 in endocrine responsiveness and how IRF1 affects the molecular signaling that regulates cell fate. By understanding the contribution of IRF1 to cellular growth and tumor suppression, we will further our knowledge on the signaling pathways of malignant diseases, which could lead to the development of novel and more effective therapeutic strategies for ER+ breast cancers. 

## 3. IRF1

### 3.1. Structure of the IRF1 Gene/Protein

IRF1 was initially characterized for its role in the transcriptional activation of type I interferon (IFN) genes. During a study on the regulation of the *IFN*-*β* gene by a virus, a factor that was called IRF1 was found to bind to the *IFN*-*β* gene promoter and to regulate its transcription. Ten splice variants of IRF1 have been identified and are labeled as splice patterns 1–10 [18]. IRF1 is now recognized as an essential player in many facets of the immune response and oncogenesis [19]. Since the discovery of IRF1 in 1988, there are now nine known IRF family members in humans and mice: IRF1, IRF2, IRF3, IRF4 (also known as PIP, LSIRF, or ICSAT), IRF5, IRF6, IRF7, IRF8 (ICSBP), and IRF9 (ISGF3*γ*) [19]. Each IRF contains a well-conserved N-terminal DNA-binding domain (DBD) of approximately 120 amino acids and five conserved tryptophan repeats [20]. The IRF DBD has a helix-turn-helix architecture that recognizes a specific DNA sequence corresponding to the IFN-stimulated response element (ISRE; G(A)AAAG/CT/CGAAAG/CT/C) [21]. 

A single point mutation (P325A) in the C-terminal region of IRF1 (multifunctional-1; Mf1; residues 301–325) increases both IRF1's ability to regulate its own transcription and rate of degradation [22]. We have also reported a novel single nucleotide polymorphism in the IRF1 gene (A4396G). IRF1-A4396G is more frequent in human breast cancer cell lines than in the general population and is more frequently expressed in African American than Caucasian women [23]. 

**Figure 1:**
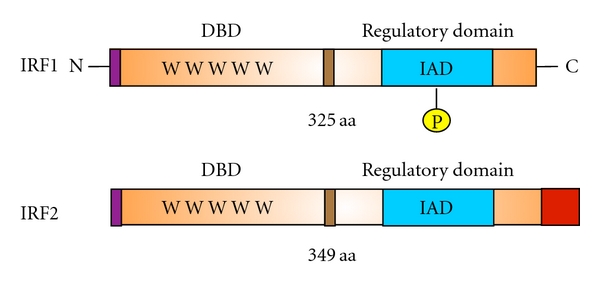
Domain structures of IRF1 and IRF2. IRF1 and IRF2 are composed of a DNA-binding domain (DBD; N-terminus) and a regulatory domain (C-terminus). The DBD is characterized by five tryptophan residues each separated by 10–18 amino acids. IRF1 and IRF2 also contain an IRF association domain (IAD). For IRF1, activity depends on phosphorylation, whereas IRF2 contains a repression domain (red). The size of each IRF1 (in amino acids; aa) is also indicated. C: carboxyl terminus; N: amino terminus.

Subsequent to the identification of IRF1, a structurally similar molecule, IRF2, was isolated by its ability to cross-hybridize with the IRF1 cDNA (Figure 1) [24]. The two factors show 62% homology in their N-terminal regions (spanning the first 154 residues), whereas the rest of the family members exhibit only 25% similarity [24]. While IRF1 is characterized by an abundance of acidic amino acids and serine-threonine residues in its carboxy-terminal region (transcriptional activation domain), IRF2 is relatively rich in basic residues [24]. When activated by IFN signaling, both IRF1 and IRF2 bind to the same DNA element, known as IRF-E, which is almost identical to the ISRE [20]. Despite their similar DBDs, these two factors are functionally distinct. IRF1 mRNA is dramatically upregulated upon viral infection or IFN stimulation [24]. A high level of IRF1, in turn, results in the induction of endogenous *IFN*-*α* and *IFN*-*β* in a variety of cell types, while IRF2 represses IRF1 transcriptional activation [25]. The IRF1 protein is also very unstable (half life *∼*30 min) compared with IRF2 (half-life *∼*8 hrs) [26]. These findings suggest respective functional activator and repressor roles for IRF1 and IRF2 for the regulation of the *IFN*-*α*/*β* genes. Further studies demonstrated markedly diverse roles for IRF family members including how they contribute to the regulation of key functions in the development of immune cells and in the control of oncogenesis. 

### 3.2. Role of IRF1 in IFN Signaling

IRF1 is expressed at low levels in unstimulated cells and is activated by many cytokines including type I (IFN*α*, IFN*β*, and others) and II (IFN*γ*) interferons, tumor necrosis factor-*α* (TNF-*α*), retinoic acid, interleukin-1 (IL-1), IL-6, and viral infection [19]. Initial signaling is mediated through the Janus-activated kinase-signal transducer and activator of transcription (JAK/STAT1) pathway, leading to the activation of the IRF1 promoter by the Stat and NF-*κ*B transcription factors [20, 27]. When a signal is transduced through the IFN receptor, phosphorylated STAT1 translocates to the nucleus where it induces the transcription of primary IFN*γ* response genes [20]. *Stat1*-deficient cells no longer respond to IFN stimulation by inducing IRF1 expression [28].

### 3.3. Regulation of IRF1 Expression

As IRF1 is a short-lived protein, rapid changes in steady-state levels occur in response to stimuli such as DNA damage or viral infection [26, 29]. The precise mechanism that regulates IRF1 stability is unknown, but IRF1 is polyubiquitinated by the E3 ubiquitin ligase and then degraded by the 26S proteasome [30]. In unstressed cells, IRF1 is chaperoned by the E3 ligase and the C-terminus of heat-shock cognate (Hsc70)-interacting protein (CHIP). In stressed cells, a complex forms between CHIP and IRF1, leading to an increase in ubiquitination of IRF1 and a decrease in its steady-state levels [31]. Additionally, the C-terminal region of IRF1 (Mf1) was identified as the regulatory domain that modulates target gene expression and determines the rate of IRF1 protein degradation. Without this enhancer region, the IRF1 protein becomes more resistant to both degradation and ubiquitination in proliferating cells [29]. IRF1 is also serine phosphorylated by casein kinase II (CKII), protein kinase A (PKA), and protein kinase C (PKC) at two clustered regions (between amino acids 138–150 and 219–231), which may also have an effect on IRF1 regulation [32].

IRF1 protein turnover and activation is also regulated by its multifunctional Mf1 domain. Recruitment of heat-shock protein (Hsp70) to the Mf1 domain leads to the further recruitment of Hsp90, which results in an increase in endogenous IRF1 protein [33]. IRF1 is a member of a class of proteins considered to be unstructured [34], which allows it to interact with multiple proteins [35]. In addition to nucleophosmin (NPM1), which can inhibit IRF1 function, the interaction between IRF1 repressors, YB-1 (Y-box protein) and TRIM28 (tripartite motif-containing 28), which are both overexpressed in various cancer types, has also been reported [35]. The presence of multiple IRF1 regulating proteins and its short protein half life suggest the presence of several redundant regulatory interactions, often the mark of a central functional activity for the control of critical cellular functions. Perhaps this is not surprising, given IRF1's ability to affect cell fate decisions.

### 3.4. Biological Functions of IRF1

IRF1 has remarkable functional diversity and controls the transcription of genes involved in mediating antiviral, immunomodulatory, and antiproliferative effects [19]. Events downstream of IRF1 activation include changes in major histocompatibility complex (MHC) class I and interferon expression, inducible nitric oxide synthase (iNOS) expression, the development of CD8^+^ T cells, induction of IL-12 and T helper differentiation, and natural killer (NK) development [36]. In addition to having critical functions in the development and activation of immune cells, IRF1 is also involved in cell cycle regulation and apoptosis in response to a variety of stressors [17]. For instance, IRF1 coordinates expression of the immunoproteasome [37], regulates human telomerase activity [38, 39], and controls vital aspects of DNA damage repair [40, 41]. IRF1 can regulate signaling that leads to the induction of apoptosis [42], which it can achieve with or without the induction of the cell cycle inhibitors, p21^cip1^ [17] or p27^kip1^ [43], and through the activation of caspases (CASP)-1 [40], CASP-3 [44], CASP-7 [45, 46], CASP-8 [44, 46], and/or Fas ligand [47]. IRF1 also induces apoptosis in a p53-dependent or-independent manner [40, 42]. Thus, IRF1 is capable of functioning in multiple cellular contexts. For example, p53 is frequently mutated in many cancers including 30% of breast cancers, but this loss of p53 function does not necessarily abrogate IRF1's capacity to regulate cell fate decisions [48].

## 4. IRF1 in Tumor Suppression

### 4.1. IRF1 as a Potential Tumor Suppressor

A critical facet of IRF1's function in host defense is the regulation of oncogenesis. The first studies to highlight IRF1's role in tumor suppression and cell cycle control were established using *IRF1 *−/− mouse embryonic fibroblasts (MEFs). *IRF1 *−/− MEFs are deficient in their ability to undergo DNA-damage cell cycle arrest, a phenotype similar to that observed in MEFs lacking the tumor suppressor p53 [17]. Furthermore, transcriptional induction of p21^cip1^ following gamma irradiation is dependent on both IRF1 and p53 [17]. Several other reports have also elucidated the involvement of IRF1 in cell growth effects [49, 50]. IRF1 can eliminate precancerous cells through apoptosis induced by DNA damage or other stimuli. For instance, wild-type MEFs require two or more oncogenic mutations for transformation, whereas a “single hit” with c-Ha-ras induces transformation in *IRF1 *−/− MEFs [51]. In this situation, apoptosis is dependent upon both p53 and IRF1. In contrast, DNA damage-induced apoptosis in mitogenically activated mature T lymphocytes is dependent on IRF1 but independent of p53 [40]. These studies demonstrate that IRF1 can mediate two crucial compounds of neoplastic progression: apoptosis and cell cycle arrest [44, 51]. 

### 4.2. IRF1 in Breast Cancer

The IRF1 locus at chromosome 5q31.1 was initially reported to be lost in a substantial proportion of leukemia and preleukemic myelodysplasia cells [52]. IRF1 is also frequently deleted (loss of heterozygosity) in esophageal [53] and gastric carcinoma (point mutation) [54]. Approximately 11% of sporadic breast cancers exhibit the loss of chromosome 5q12–31, the most frequent chromosome loss detected by comparative genomic hybridization (CGH) [55]. Other studies report that approximately 30% of neoplastic breast tissues have loss of IRF1 by immunohistochemical staining when compared with normal breast epithelium [56]. Furthermore, high-grade ductal carcinoma *in situ* (DCIS) or node-positive invasive ductal cancers were less likely to express IRF1 and were much more likely to have higher oncogenic IRF2 protein than normal [56]. CGH also implicates IRF1 loss of heterozygosity (LOH) in 50% of BRCA1 mutated breast cancer tumors [57, 58]. These results are consistent with the hypothesis that loss of IRF1 expression in some breast cancers contributes to the loss of appropriate growth control. 

Allelic loss of IRF1 is detected in 32% of women with breast cancer (12/37 breast tissue specimens) [59]. This loss of heterozygosity is associated with an increased risk of recurrence and risk of death in the cases studied, strongly implicating a tumor suppressive role for the *IRF1 *gene in breast cancer [59]. Analyses of two publically available ONCOMINE cancer gene microarray datasets also imply an important tumor suppressive role for *IRF1* in many sporadic breast cancers. Protein expression studies show that in breast tumors the most prevalent location of IRF1 is within the cytosol (90%); this location suggests a transcriptionally inactive form of IRF1 [60]. In contrast, 51% of the reported tumors expressed IRF1 in the nucleus (in more than 50% of the tumor cells), consistent with a potential to represent a transcriptionally active form [60]. Thus, some breast tumors may differentially regulate the activation of IRF1 by controlling its subcellular localization. These observations are consistent with a study reporting higher levels of IRF1 protein in adjacent normal breast epithelium when compared with high-grade DCIS or lymph node-positive invasive ductal carcinoma of the breast [58]. Collectively, these studies imply that some cells may bypass the growth inhibitory mechanisms of IRF1 by down-regulating its expression [60, 61]. This observation is supported by the evidence that reduced IRF1 expression in breast cancer cells is associated with low caspase activity, low apoptosis, and ultimately, increased cell survival [16, 44, 62].

## 5. IRF1 in Regulating Antiestrogen Resistance

**Figure 2:**
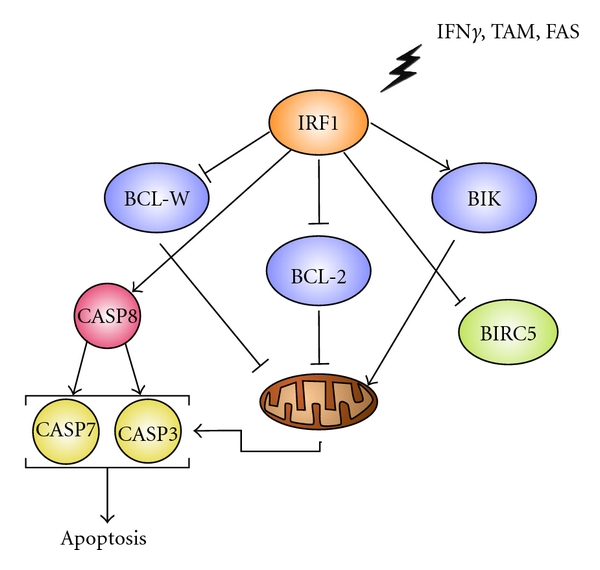
Putative IRF1-driven cell fate through BCL2 family members, BIRC5, and caspases.

IRF1 is a key mediator of cell death for the antiestrogens fulvestrant [63] and tamoxifen [64]. The pathway described in Figure 2 illustrates the mechanism by which IRF1 modulates apoptosis. MCF7 and T47D breast cancer cells expressing a dominant negative IRF1 (dnIRF1), which lacks the carboxyl-terminal transcriptional activation domain, do not undergo FAS-induced cell death and are, in turn, less sensitive to AEs [63]. Moreover, dnIRF1 expressing cells result in the inhibition of CASP3/7 and CASP8, indicating the primary antitumorigenic role of IRF1 is through the promotion of apoptosis [44]. 

Furthermore, breast cancer cells that have acquired resistance to FAS show significant upregulation of microRNA (miRNA)-221 and -222 expression [65]. Overexpression of these two miRNAs affects several oncogenic signaling pathways, including the JAK/STAT and p53 pathway, supports ER*α*-independent proliferation, and promotes tumor progression [65]. Thus, miRNA-221/222 may serve to down-regulate the tumor suppressing effects of IRF1 in breast cancer cells following antiestrogen therapy. An acquired resistance model involving aromatase inhibitors shows that IRF1 expression is reduced in long-term estrogen deprived MCF7 cells, indicating IRF1 is a key gene that is consistently reduced in AE resistant breast tumors [66].

In ER+ breast cancer cells, Bouker et al. have shown that IRF1 signaling reduces the rate of cell proliferation and the incidence of human breast cancer xenografts in athymic nude mice [44]. A dnIRF1 blocks the effect of parthenolide, a small molecule inhibitor of NF-*κ*B, and synergistically interacts with antiestrogens *in vitro* to reverse the antiestrogen-resistance phenotype [67]. Moreover, antiestrogen resistant breast cancer cells MCF7/LCC9 have greater BCL2 protein expression compared to antiestrogen sensitive breast cancer cells (MCF7/LCC1) [68]. LCC9 cells are more dependent on BCL2 for their survival, since they exhibit a significantly greater growth inhibition by the small molecule BCL2 inhibitor HA 14-1 when compared with their LCC1 controls, and the LCC9 antiestrogen-resistant cells also express lower IRF1, indicating that a clear functional link exists between IRF1, NF-*κ*B activation, BCL2 expression, and ER+ breast cancer cell fate [62, 68].

Functionally relevant interactions between IRF1 and at least two different members of the epidermal growth factor receptors (EGFR) superfamily have been reported. EGFR induces expression of IRF1 [69], and IRF1 also can regulate EGFR expression [69], suggesting a possible role of IRF1 in EGFR overexpressing breast cancers. The coexpression of EGFR and IRF1 appears key for the induction of apoptosis, and blocking STAT5*α* with a dominant negative eliminates the transcriptional synergy and ability of IRF1 and EGFR to induce apoptosis [70]. Overexpression of EGFR (HER1) is common in the subgroup generally referred to as “triple negative breast cancer” (TNBC); these tumors lack the ER, the PgR, and have normal human epidermal growth factor 2 receptor (HER2/neu) levels. TNBCs are particularly challenging to treat due to the lack of targeted therapy. Thus, expression of IRF1 and the ability of EGFR:IRF1 to affect cell death may be important in TNBC. 

 IRF1 may play an important role in another breast cancer subgroup, the HER2-amplified breast cancers. HER2 overexpressing breast cancer has a generally poor prognosis, with approximately 35% of cases responding to the HER2-targeted therapy, Herceptin [71]. The importance of interactions between IRF1 and HER2/neu are less well described. For example, IRF1 expression becomes constitutively activated in 3T3 cells transduced with HER2/neu [72]. IFN*γ* induces IRF1 in MMTV-neu transgenic mice and leads to apoptosis [73]. Several observations have led to the hypothesis that breast cancer patients with HER2/neu overexpression may benefit less from TAM than those whose tumors with low expression of this oncogene. For example, TAM acts similarly to estrogen withdrawal in MCF7 cells transfected with the HER2/neu oncogene [74]. However, the true relationship remains controversial, since a recent meta-analysis found no association between TAM responsiveness and HER2/neu expression. In contrast, a benefit for both anthracycline and taxane-based polychemotherapies shows a clear association with Her2/neu status [75]. Given the close relationship between IRF1 and EGFR family members, studies of the role of IRF1 in modifying responsiveness to taxanes and anthracyclines in the context of triple negative and Her2/neu breast cancers seem warranted.

## 6. IRF1 Induces Cell Death through Apoptosis

Cell signaling to affect an irreversible cell death results in one or more forms of cell death including apoptosis (programmed cell death 1; PCD1), autophagy (PCD2), and necrosis (PCD3) [76, 77]. In the context of endocrine responsive breast cancers, it is important to understand the underlying mechanism that IRF1 uses to mediate tumor suppressive activity. A recent study by Ning et al. shows how combined IFN*γ* and FAS treatment increases the expression and nuclear translocation of IRF1 [62]. AE sensitivity is restored in AE-resistant breast cancer cells through an IRF1-dependent increase in mitochondrial outer membrane permeability and activation of the intrinsic apoptotic pathway [62]. Apoptosis is then executed by selected caspases including CASP7, CASP8, and CASP9 [62, 78]. Moreover, IRF1-induced apoptosis is ligand independent but requires a fas-associated death domain (FADD)/CASP8 complex that forms intracellularly [79]. Members of the TGF*β* family, which have long been implicated in endocrine resistance [80], also induce IRF1 expression that can lead to apoptosis in breast cancer cells [61]. Overexpression of IRF1 in breast cancer cells can induce apoptosis, which is consistent with its tumor suppressive activity [68]. 

BCL2 family proteins have either pro- (including BAD, BAK, BID, BAX, BID), or antiapoptotic activities (such as BCL2, BCLW, and BCLXL) and are functionally involved in the regulation of cell fate [81]. IRF1 mediates cell death, specifically apoptosis, by signaling through several BCL2 family members and caspases [62, 78]. IRF1 can increase expression of proapoptotic BAK, BAX, BIK, while decreasing expression of the antiapoptotic BCL2 and BCLW [62]. Moreover, enhanced IRF1 expression in breast cancer cells down-regulates the inhibitor of apoptosis protein, survivin (BIRC5) [82]. Reduced expression of antiapoptotic BCL2 members and survivin, with an increase in proapoptotic BCL2 protein expression, leads to an alteration in mitochondrial membrane permeability, release of cytochrome *c*, and ultimately apoptosis executed by selected caspases [62]. We propose a novel signal transduction pathway operating in breast cancer (Figure 2). In this pathway, IRF1 plays a central role in regulating expression of BCL2 members, survivin, and caspases, thus, determining the cell fate decision to live or to undergo apoptosis.

The regulation of several BCL2 family members is also dependent on NF-*κ*B, a transcription factor critical in the regulation of cell proliferation and in resistance to cytotoxic drugs [83]. NF-*κ*B is directly implicated in IFN*γ* signaling. For example, NF-*κ*B expression and activation is significantly increased in MCF7/LCC9 cells when compared to the parental, antiestrogen sensitive MCF7/LCC1 breast cancer cells; NF-*κ*B can form functional heterodimers with IRF1 that regulate the expression of genes through an IFN*γ* activation site (GAS)/kappaB promoter element [84]. Increased NF-*κ*B activation is functionally associated with conferring an antiestrogen resistant phenotype, which it does in part by modulating CASP8 activity, mitochondrial function, and apoptosis. Interestingly, the activities of NF-*κ*B appear to act by increasing BCL2 expression [85, 86], whereas this expression is inhibited by IRF1; endogenous IRF1 expression is reduced in these cells indicating that the balance between IRF1 and NF-*κ*B may be critical in determining cell fate. These activities are likely independent of IRF1:NF-*κ*B heterodimer formation [87], unless the primary role of these in the endocrine resistant phenotype is to sequester IRF1. The upregulation of NF-*κ*B expression in resistant cells would likely leave sufficient NF-*κ*B, in excess of that bound to IRF1, free to regulate its prosurvival signaling. 

The combination of IFN*γ* and FAS reduces NF-*κ*B protein expression and transcriptional activation through the induction of IRF1 [62]. In addition to NF-*κ*B, NPM1 is also significantly overexpressed in MCF7/LCC9 cells [16], and NPM1 binds and inhibits IRF1 function [88]. Thus, NPM1 may act by blocking/eliminating a caspase cascade in breast cancer cells through its ability to reduce IRF1 and signaling to apoptosis [16].

STAT1, a transcription factor upstream of IRF1 and mediator of IFN signaling, is also considered a tumor suppressor gene [89]. Protein levels of STAT1 and phosphorylated STAT1 are substantially increased with IFN*γ* treatment, leading to an increase in IRF1 [62, 90]. Moreover, *Stat1 *and *IFN* *γ* receptor null mice show spontaneous tumor growth when either exposed to methylcholanthrene, or are bred into a *p53*-deficient background [91]. STAT1 also has a tumor suppressive role in mammary epithelium in ERBB2/neu-induced breast cancer [89]. These studies provide greater insight into the cell-specific roles of IFN and STAT1 signaling, ultimately effecting IRF1 induction. 

## 7. Clinical Implications

The ability of interferons to sensitize breast cancer cells to TAM was shown over 20 years ago. Van den Berg et al. showed that the combination of IFN*α* and TAM had a greater antiproliferative effect on ZR-75-1 breast cancer cells than either drug alone [92]. IFN*α*, IFN*β*, and IFN*γ* have each been used in the treatment of breast cancer either to induce antiestrogen sensitivity and/or stimulate cellular immunity [93]. Pilot studies suggest that IFN*β* can improve clinical benefit and/or overall survival in patients with metastatic breast cancer and will be tested in phase III clinical trial [93]. Although mixed results have been shown with IFN*α* as an antitumor agent, IFN*β* and IFN*γ*, alone or combined with an antiestrogen, have shown to be effective in increasing hormonal dependency and overcome TAM resistance [94]. The pegylation (covalent attachment of polyethylene glycol molecule to a protein) of IFN*α* also induces IRF1-mediated signaling and sensitizes melanoma cells to chemotherapy [95]. Pegylated IFN*α* is currently being used in monotherapy [96] and in combination with chemotherapy for several different types of cancers [97, 98].

Restoring IRF1 expression or controlling its modulation may be useful for the treatment of ER-positive breast tumors that have acquired resistance to endocrine therapy. Therefore, a clear understanding of IRF1 signaling and how it contributes to cell death is necessary for the discovery of new drug candidates and better predictors of how tumors will respond to endocrine therapy.
